# Does Age, Residency, or Feeding Guild Coupled with a Drought Index Predict Avian Health during Fall Migration?

**DOI:** 10.3390/ani12040454

**Published:** 2022-02-12

**Authors:** Jenna E. Stanek, Brent E. Thompson, Sarah E. Milligan, Keegan A. Tranquillo, Stephen M. Fettig, Charles D. Hathcock

**Affiliations:** 1 Los Alamos National Laboratory, Los Alamos, NM 87545, USA; bthompson@lanl.gov (B.E.T.); hathcock@lanl.gov (C.D.H.); 2 Bandelier National Monument, Los Alamos, NM 87544, USA; sarah_milligan@nps.gov (S.E.M.); ktranquillo@hotmail.com (K.A.T.); 3 US Fish and Wildlife Service, Sacramento, CA 95825, USA; osprey@cybermesa.com

**Keywords:** avian mortality, drought, avian health, climate, bird banding, migratory birds, insectivore

## Abstract

**Simple Summary:**

After a large avian mortality event occurred in New Mexico in the fall of 2020, we performed an analysis using 11 years of fall bird banding data at two locations in north-central New Mexico to investigate the influence of drought on avian health. Carcass studies after the event indicated that starvation was the primary cause of death. Using fall bird banding data along with regional drought indices, we used multiple logistic regression to predict the body health conditions of a bird during the fall migration period. We found that fat scores for younger, insectivorous, migratory birds were less likely to be greater than zero as drought severity levels increased. Our results suggest that migratory insectivores in the southwestern United States may be less resilient to drought-related climate change.

**Abstract:**

Birds are good indicators of environmental change and are often studied for responses to climate. Many studies focus on breeding birds, while fewer look at the migration period, which is a critical time for many birds. Birds are more susceptible to unusual climatic events during their migration due to the metabolic stress of long-distance movements. In the fall of 2020, an unusual cold weather event coupled with drought and wildfire smoke led to a large avian mortality event in New Mexico. Later analysis pointed to the mortality being largely due to starvation. This was the impetus for our research. We used 11 years of fall bird banding data from two locations, along with local drought indices, to determine what predicts avian health during the migration period. We used fat score data from over 15,000 individual birds to assess whether drought indices, age, diet, or residency influenced avian health using multiple logistic regression. We found that the probability of positive fat scores decreased as drought severity increased for younger, insectivorous, migratory birds. Insectivores had a higher probability of receiving a fat score greater than zero relative to local drought conditions, which is important, since many North American insectivores are in steep decline. Migratory birds showed a greater response than year-round residents, and older birds showed a lower but significant response compared to hatch-year birds. Our results suggest that migratory insectivores in the southwestern United States may be less resilient to drought-related climate change.

## 1. Introduction

Birds are good indicators of environmental change; therefore, the status and trends of bird populations are critical for identifying and understanding environmental issues, including climate change, and for developing effective management and conservation practices. Most bird monitoring in North America is conducted during the breeding season, such as the North American Breeding Bird Survey, which is a large-scale, long-term monitoring program designed to track the status and trends of North American bird populations [1]. Another long-term monitoring effort performed during the breeding season is the Monitoring Avian Productivity and Survivorship (MAPS) program, which monitors avian vital rates [2]. Since 1989, over 1100 MAPS stations have been operated for at least one year [3].

Far less research is available on how climate change may be affecting birds during the fall migration [4]. Migration is a critical time in a migratory bird’s life cycle where they are pushed to the edge of their physiological limits [5] and can experience the largest amount of annual mortality [6,7]. Some federal agencies and other organizations document the fall migration patterns of passerines to monitor the status and trends of resident and migratory bird populations. Counts and captures of spring and fall migrants generate useful information on the status and trends of source populations [8]. Many banding stations collect fall bird banding data, though there is not an equivalent level of standardization as seen with MAPS. Recent research [9] analyzed spring and fall banding data from 1969 to 2015, and found that an overall decline in numbers is present, but abundance was not able to be tied to several of the life history traits examined. Most spring and fall data are often used to examine migration timing [10,11,12]. For example, McKinnon et al. [13] examined life history traits during fall migration for a single species using geolocator data, and Rousseau et al. [14] used fall banding data to assess the demographics of the Rufous Hummingbird (*Selasphorus rufus*). Body condition estimates, often using the amount of fat in a migrating bird, have been used to assess fall migration timing in relation to weather [15,16,17,18]. 

Unusual climatic events are becoming more frequent in nature and severity [19]. For example, in February 2021, unprecedented cold weather hit southern Texas [20], and in the fall of 2020, a cold weather event preceded by poor summer precipitation led to a large bird die-off in New Mexico [21]. The die-off was attributed to poor air quality from wildfire smoke on North America’s west coast and extreme fluctuating weather patterns, with record highs on September 6th and 7th, followed by record early snow on September 9th, and record cold for the season on September 9th and 10th [21]. Some estimates suggest that upwards of one million birds died in this event across five states in the southwestern United States [22]. Many of the birds were collected and submitted to the United States Geological Survey’s National Wildlife Health Center to determine the cause of death. The only consistent impacts to all of the birds were a high level of breakdown of muscle tissue in the breast meat, low or nonexistent fat, and general emaciated conditions [21]. With the decreased fat reserves, most of these birds likely died from hypothermia. Some research has already been performed to look at the 2020 avian mortality event in more detail. Yang et al. [23] examined spatiotemporal patterns at a high level, and suggested that birds that were already stressed because of drought conditions could be more prone to other factors, including distance to wildfire and air quality.

In north-central New Mexico at Los Alamos National Laboratory (LANL) and the adjacent Bandelier National Monument (BNM), fall bird banding operations have occurred regularly since 2010. Because this large mortality event occurred during our fall bird banding season in 2020, we were interested to see if our bird condition data would reflect the conditions identified by the National Wildlife Health Center. We hypothesized that insectivorous migratory birds would be most affected by drought conditions. We were interested to see if more severe local drought conditions could be associated with a greater probability of a fat score of “none”, and if migratory insectivorous birds are more at risk to increased drought severity. If so, then drought conditions beyond a certain threshold may predict future die-offs of this nature. We set out to answer the question: What predicts fat based on our fall banding stations and drought index data?

## 2. Materials and Methods

### 2.1. Site Location

We conducted this study at two locations in north-central New Mexico, with one in a large wetlands complex at LANL, and another at a higher elevation site in BNM. The fall migratory banding stations were started with the goal to learn about the species diversity and quantity of birds using these sites during their fall migration period, which is a critical part of a bird’s annual life cycle. The fall migration monitoring site in BNM is located at the Alamo Boundary Trailhead (35.83343° N, −106.44348° W, WGS 84, elevation: 2717 m) in the northwestern area of BNM. The banding station consists of 20 mist-nets (30 mm mesh) that are 12 m long and 2.5 m in height. The area is composed of ponderosa pine (*Pinus ponderosa*), aspen (*Populus tremuloides*), and Douglas fir (*Pseudotsuga menziesii*). The fall migration monitoring site at LANL consists of 14 mist-nets of the same design, which are deployed in the upper end of the Pajarito wetlands complex (35.83608° N, −106.25545° W, WGS 84, elevation: 2038 m). This wetlands complex is composed of narrowleaf cottonwood (*Populus angustifolia*), narrowleaf willow (*Salix exigua*), and broadleaf cattail (*Typha latifolia*). These locations occur near the overlap of the Pacific and Central migratory bird flyways [24] and migratory birds likely come from both flyways into the study area. 

### 2.2. Data Collection

Banding operations took place between August and October, from 2010 to 2020. Net locations were placed strategically to maximize the number of birds captured. The size of the band followed the requirements in the Bird Banding Manual [25]. Captured birds were identified, measured, and banded with a Federal Bird Banding Laboratory migratory bird band. All birds were identified, aged, sexed, weighed, measured, fat-scored, and checked for signs of molt. The aging and sexing criteria were based on Pyle [26]. Fat scores were based on the standardized categories set forth by the Institute for Bird Populations and include “none”, “trace”, “light”, “half”, “filled”, “bulging”, “greatly bulging”, and “very excessive” [2]. Fat scores provide a more direct measure of fuel stores than measures based on body mass [18], which is known to fluctuate seasonally independently of fat scores [27]. 

### 2.3. Statistical Analyses

We used multiple logistic regression models to assess the influence of drought indices, age, diet, and residency on avian health (Table S1). We aimed to assess the relative health of birds based on fat score; therefore, we grouped the response variable of fat score as either “none”, which was equal to 0, and all other fat categories were grouped together and made equal to 1. We chose to group the data in this manner to avoid zero-inflation problems in the statistical analyses and to evenly distribute the response variable terms, because approximately half of fat scores were categorized as “none”. Studies have shown that fat scores throughout the annual life cycle are higher during fall migration [18,28]. We, therefore, hypothesized that a migrant could be deemed as in better physiological condition if they had a fat score greater than zero during fall migration. We used the R statistical software for all data analyses [29]. 

**Figure 1:**
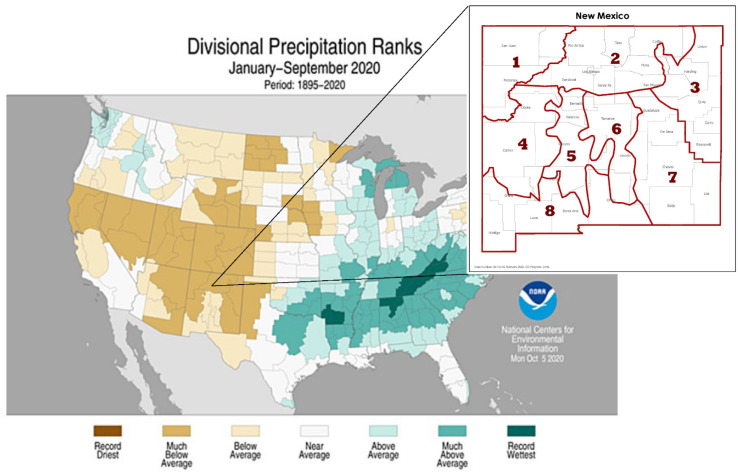
Precipitation ranks from January through September 2020 for the United States climate divisions [35], with an inset map showing the New Mexico climate divisions.

We used the information-theoretic approach to rank a priori models with Akaike’s Information Criterion (AIC), the difference between each model compared to the model with the lowest AIC (ΔAIC), and Akaike weights [30]. The candidate model set was based on combinations of variables hypothesized to contribute to avian health. Highly correlated variables with a variance inflation factor >5 were not included in the same model [31] or candidate set of models. We used deviance residual goodness-of-fit tests to check the global model for over-dispersion. We selected the most parsimonious model ΔAIC ≤ 2wi > 0.9 [30] and calculated 95% confidence intervals of the odds ratio (OR); confidence intervals not containing 1 indicated conclusive estimates [32]. We excluded data from birds captured during the same year because we wanted to compare fat scores between the years. We removed “rare” species caught ≤ 3 times from the 10-year dataset. Additionally, we grouped subspecies into species categories to standardize the data for further analyses. For example, different junco races were all considered to be dark-eyed juncos (*Junco hyemalis*) for this dataset. We grouped birds into one of three diet classifications based on life history information available from Cornell’s *The Birds of North America Online* [33]. The three feeding groups were 1) granivores, where diet consists primarily of seeds; 2) insectivores, where diet consists primarily of insects; and 3) omnivores, where the diet is split between the two. We categorized bird species as either breeding in the area or migrants, based on our own records and regional field guides. The Palmer Drought Severity Index (PDSI) uses readily available temperature and precipitation data to estimate relative dryness [34]. It is a standardized index that spans from −10 (dry) to +10 (wet), and has been reasonably successful at quantifying long-term drought. PDSI data are all publicly available. We hypothesized that avian health would partly be influenced by drought severity where PDSI values: 0.0 to −0.5 = normal; −0.5 to −1.0 = incipient drought; −1.0 to −2.0 = mild drought; −2.0 to −3.0 = moderate drought; −3.0 to −4.0 = severe drought; <−4.0 = extreme drought [34]. We used PDSI data from New Mexico, climate division 2, from the National Centers for Environmental Information public database [35] to capture local drought conditions (Figure 1).

Of the most common migratory bird species in our dataset (*n* ≥ 50), 19 of 22 were species reported in the 2020 mortality event, while 11 of 12 were year-round bird species (Table 1). The Wilson’s Warbler (*Cardellina pusilla*) was the second most common migrating insectivore in our dataset (*n* = 1181) and the most common bird reported in the 2020 Southwest Avian Mortality Project under iNaturalist: https://www.inaturalist.org/projects/southwest-avian-mortality-project (accessed on 15 December 2021).

**Figure 2:**
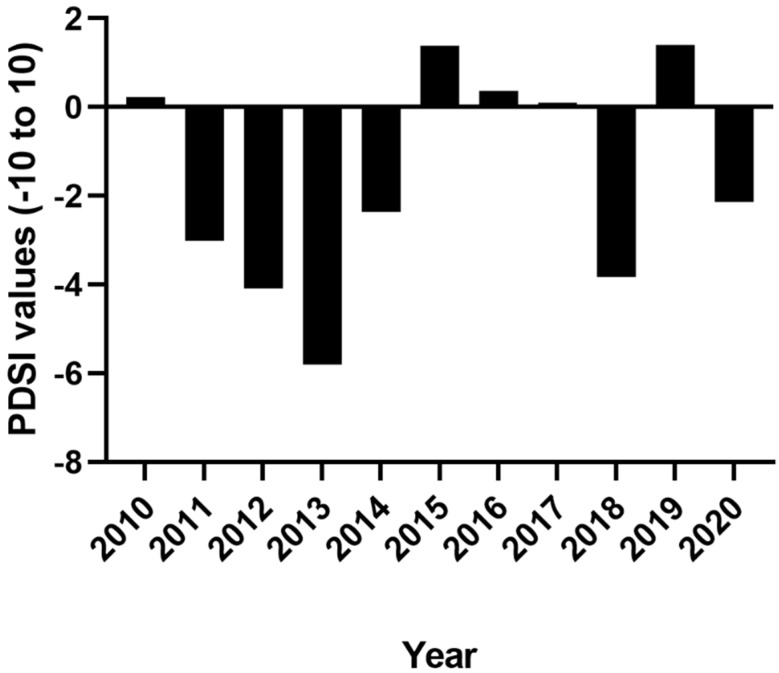
Averages from December 2009 through August 2020 for New Mexico climate division region 2 Palmer Drought Severity Index (PDSI); negative values indicate drier conditions and positive values indicate wetter conditions.

For analyses, we used averages of local (New Mexico climate division region 2) PDSI data [35] from December through August from 2009 to 2020 (Figure 2). We investigated avian health, measured as having some quantity of fat or not having any fat at all, in relation to local drought severity, residency, different feeding groups, and age (hatch year versus after hatch year).

## 3. Results

Banding operations took place between August and October, from 2010 to 2020. Our analyses examined factors that we hypothesized to have an effect on fat scores during fall migration, with a total 15,244 individuals from 71 species, of which 44 species were considered to be migrants. Our top-ranked model with the PDSI from the New Mexico region 2 climate division had overwhelming support (i.e., wi > 0.9; Table 2) and included age and interaction terms for diet and resident.

**Figure 3:**
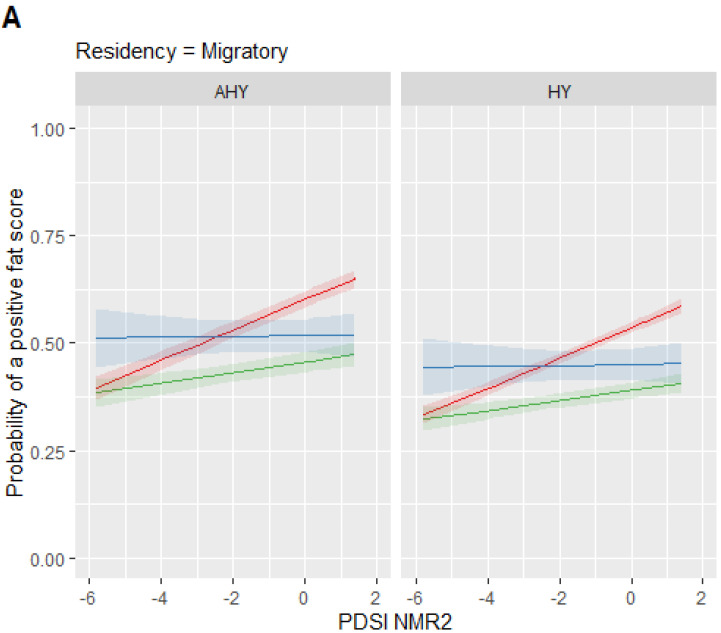

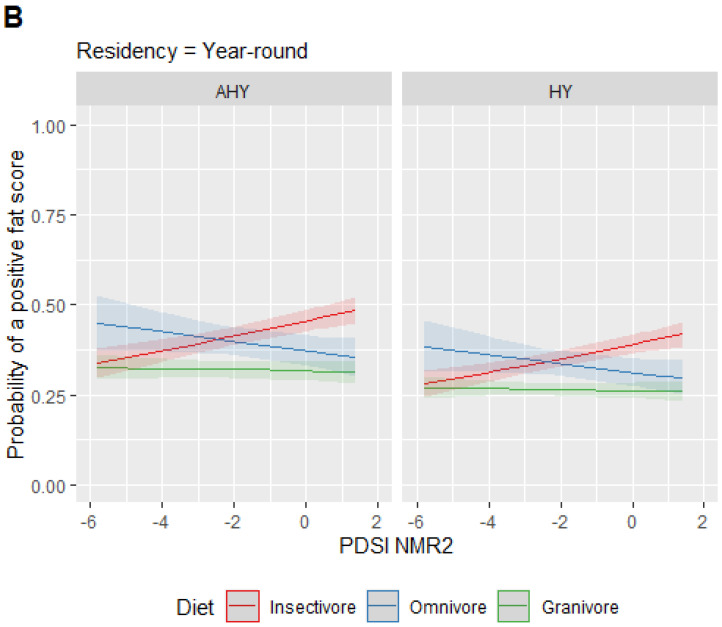
Probability of receiving a positive fat score based on (**A**) migratory versus (**B**) year-round residency for age (AHY = after hatch year, HY = hatch year) and diet (insectivore, granivore, and omnivore) in relation to the Palmer Drought Severity Index for New Mexico region 2 (NM region 2).

The top model had no competing models within ΔAIC < 2 (Table 2). We found no significant evidence of over-dispersion nor under-dispersion. The top model contained the variables PDSI, diet, residency, and age with interaction effects of PDSI for residency and diet. The top model results show that migratory birds (Figure 3A) had a higher probability of receiving a fat score greater than zero in wetter conditions (when PDSI values increased) compared to year-round residents (OR 1.06, 95% CI: 1.02–1.10; Figure 3B). Insectivorous birds also had a higher probability of receiving a fat score greater than zero as local PDSI values increased (OR 1.10, 95% CI: 1.07–1.13; Figure 3) compared to omnivores and granivores, which were not affected by local drought conditions (Figure 3). Both ages were affected by local drought, but hatch-year birds had a lower probability of receiving a positive fat score compared to after-hatch-year birds (OR 0.76, 95% CI: 0.71–0.83; Figure 3). For example, for a hatch-year, migratory, insectivorous bird, the probability of a positive fat score was 0.59 (95% CI = 0.57, 0.60) when the New Mexico region 2 PDSI is 1.41, which was almost double that of the probability (0.33 (95% CI = 0.31, 0.35)) during an extreme drought when New Mexico region 2 PDSI is −5.80. Other combinations of characteristics and PDSI values can be evaluated using the probabilities shown in Figure 3. 

## 4. Discussion

Teasing out impacts to avian communities from global climate change remains a challenging endeavor because many ecological processes are impacted. Many studies model impacts based on variables where we have data, such as temperature or rainfall, but other indirect impacts from climate change, such as smoke from wildfires or the effects on the timing of food resources, are harder to quantify. Here, we try to quantify bird fitness, measured by fat scores, in relation to local PDSI values. Coupled with the ecological values of the birds, including their age, diet, and migratory status, we found support for our hypothesis that we can predict body health indices before the fall migration peaks. 

Insectivores had a higher probability of a fat score greater than zero relative to local drought conditions, while granivores and omnivores showed a non-significant and relatively flat relationship. This is important because, in North America, we are seeing up to a 31% decline in aerial insectivores [36]. Drought may exacerbate the availability of insects within the same year. Insect hatch and survival is dependent on winter and spring moisture of the same year, whereas seed production is more frequently associated with plant resources, which may be years in the making [37]. For example, Parmenter et al. [38] found that two-needle pinyon (*Pinus edulis*) seed production was positively related with total precipitation on a 1-year lag. 

For our study, migratory birds showed a greater predictive response for higher fat scores than resident birds in relation to local drought conditions. This follows the logic that migratory birds need to maintain fat reserves to power their migration, where local resident birds do not. Physical exertion without nourishment to support recovery leads to conditions observed during the 2020 avian mortality event [21,39]. When we looked at the age of the birds for a predictive response for fat scores, both ages were affected by local drought, but hatch-year birds had a lower probability of receiving a positive fat score than after-hatch-year birds. 

## 5. Conclusions

Starvation due to drought and insect decline may make migrants more susceptible to wildfire smoke and extreme weather events, especially young birds that lack experience in migration pathways. Unseasonal cold snaps are not unprecedented in the southwestern United States. In September 2006, there was a cold snap, with a low of 4.61˚C, with no documented avian mortality compared to the 2020 cold weather event, which had a low of 5.6 ˚C and thousands of reported avian deaths (retrieved 11 December 2021 from the Wildlife Health Information Sharing Partnership event reporting system online database, https://whispers.usgs.gov/home). This would suggest that birds are in poorer health, cannot refuel as quickly, and are being affected by increased anthropogenic influences (wildfire, habitat loss, climate change), more so than they were in 2006 during a similar event. As the climate changes across already-stressed ecosystems, there is no doubt that species will be affected, but to what extent and which will be most vulnerable remain uncertain. Our results suggest that migratory insectivores in the southwestern United States may be less resilient to drought-related climate change. When and where mortality occurs throughout a migratory bird’s annual cycle may change with age and have implications for the overall population growth or decline [7]. Identifying if survivorship is lowest during migration, for a widespread declining species, would help inform management recommendations and actions. For example, if the highest mortalities for a declining species occur during specific migration routes, management could focus on increasing habitat quality, including in urban settings, within migratory pathways by creating sanctuaries of stop-over habitats for migrants to refuel. Our study provides another important piece of information for the management of migratory birds, especially in light of the rapid changes to the landscape happening in the southwestern United States.

## Figures and Tables

**Table 1:** List of species with ≥50 birds captured at fall banding sites from 2010 to 2020. Species recorded in the 2020 mortality event are indicated with an “X”; diet is indicated as I = insectivore, O = omnivore, G = granivore; residency is indicated as YR = year-round, M = migratory.

Common Name	Species	N	Mortality Event	Diet	Residency
Chipping Sparrow	*Spizella passerina*	1892	X	G	M
Dark-eyed Junco	*Junco hyemalis*	1719	X	G	M
Yellow-rumped Warbler	*Setophaga coronata*	1390	X	I	M
Wilson’s Warbler	*Cardellina pusilla*	1181	X	I	M
Pine Siskin	*Spinus pinus*	1036	X	G	YR
Ruby-crowned Kinglet	*Corthylio calendula*	907	X	I	M
Orange-crowned Warbler	*Vermivora celata*	659	X	I	M
Dusky Flycatcher	*Empidonax oberholseri*	569	X	I	M
Western Bluebird	*Sialia mexicana*	504	X	I	YR
House Wren	*Troglodytes aedon*	499	X	I	M
Lesser Goldfinch	*Spinus psaltria*	468	X	G	YR
Hermit Thrush	*Catharus guttatus*	353	X	I	M
Lincoln’s Sparrow	*Melospiza lincolnii*	345	X	O	M
Virginia’s Warbler	*Leiothlypis virginiae*	289		I	M
Hammond’s Flycatcher	*Empidonax hammondii*	282		I	M
MacGillivray’s Warbler	*Geothlypis tolmiei*	245	X	I	M
White-crowned Sparrow	*Zonotrichia leucophrys*	242	X	O	M
Western Wood-Pewee	*Contopus sordidulus*	228	X	I	M
Warbling Vireo	*Vireo gilvus*	214		I	M
American Robin	*Turdus migratorius*	177	X	I	M
Western Tanager	*Piranga ludoviciana*	165	X	I	M
Green-tailed Towhee	*Pipilo chlorurus*	146	X	O	M
Bushtit	*Psaltriparus minimus*	135	X	I	YR
Mountain Chickadee	*Poecile gambeli*	130	X	I	YR
House Finch	*Haemorhous mexicanus*	131	X	G	YR
Spotted Towhee	*Pipilo maculatus*	114	X	O	YR
Townsend’s Warbler	*Setophaga townsendi*	87	X	I	M
Brewer’s Sparrow	*Spizella breweri*	78	X	I	M
Cordilleran Flycatcher	*Empidonax occidentalis*	78	X	I	M
Northern Flicker	*Colaptes auratus*	74	X	I	YR
Pygmy Nuthatch	*Sitta pygmaea*	73	X	I	YR
White-breasted Nuthatch	*Sitta carolinensis*	62		I	YR
Williamson’s Sapsucker	*Sphyrapicus thyroideus*	54	X	I	YR
Yellow Warbler	*Setophaga petechia*	50	X	I	M

**Table 2:** Model selection results of logistic regression models for estimating fat score in relation to Palmer Drought Severity Index (PDSI) from New Mexico region 2 climate division, diet classification (insectivore, granivore, and omnivore), residency (migrant versus year-round resident) and age (hatch year versus after hatch year); data collected from 2010 to 2020. Model selection based on Akaike’s Information Criterion (AIC), and difference in AIC between each model compared to the model with the lowest AIC (ΔAIC).

Model	K ^a^	AIC ^b^	ΔAIC ^c^	*w*_i_
PDSI × Diet + PDSI × Resident + Age ‡	9	20208.64	0.00	1.000000
PDSI + Resident + Age + Diet	6	20280.51	71.87	0.000000
PDSI + Resident + Diet	5	20320.66	112.02	0.000000
PDSI × Diet + Age	7	20343.18	134.54	0.000000
PDSI + Age + Diet	5	20394.89	186.25	0.000000
PDSI x Resident + Age	5	20408.49	199.85	0.000000
PDSI + Resident + Age	4	20423.72	215.07	0.000000
PDSI + Diet	4	20424.61	215.96	0.000000
PDSI + Resident	3	20456.59	247.94	0.000000
Age + Diet + Resident	5	20460.35	251.71	0.000000
Diet + Resident	4	20508.08	299.44	0.000000
PDSI + Age	3	20601.37	392.73	0.000000
PDSI	2	20621.07	412.42	0.000000
Diet + Resident	3	20630.92	422.28	0.000000

^a^ Number of model parameters. ^b^ Akaike’s Information Criterion (AIC). ^c^ Difference between AIC_c_ of model and AIC_c_ of top-ranked model. ‡ indicates the selected top model.

## Data Availability

Publicly available climate datasets were analyzed in this study. These data can be found here: https://www.ncdc.noaa.gov/cag/ (accessed on 10 February 2022).

## Supplementary Materials

The following supporting information can be downloaded at: https://www.mdpi.com/article/10.3390/ani12040454/s1, Table S1: Bird Banding Data 2010–2020.
